# Estimation of *Mycobacterium avium* subsp. *paratuberculosis* load in raw bulk tank milk in Emilia‐Romagna Region (Italy) by qPCR

**DOI:** 10.1002/mbo3.350

**Published:** 2016-03-17

**Authors:** Matteo Ricchi, Roberto Savi, Luca Bolzoni, Stefano Pongolini, Irene R Grant, Caterina De Cicco, Giulia Cerutti, Giuliana Cammi, Chiara A. Garbarino, Norma Arrigoni

**Affiliations:** ^1^ Istituto Zooprofilattico Sperimentale della Lombardia e dell'Emilia‐RomagnaNational Reference Centre for ParatuberculosisStrada Faggiola 1, loc.Gariga ‐ Podenzano (PC)29027Italy; ^2^ Istituto Zooprofilattico Sperimentale della Lombardia e dell'Emilia‐RomagnaCentro di Referenza Nazionale per i Rischi Emergenti in Sicurezza Alimentare20133MilanItaly; ^3^ Risk Analysis UnitIstituto Zooprofilattico Sperimentale della Lombardia e dell'Emilia‐RomagnaVia dei Mercati 13/AParma43121Italy; ^4^ Institute for Global Food SecuritySchool of Biological SciencesQueen's University Belfast97 Lisburn RoadBelfastBT9 7BLNorthern IrelandUnited Kingdom

**Keywords:** Bulk tank milk, *Mycobacterium avium* subsp. *paratuberculosis*, peptide magnetic separation, qPCR, quantification

## Abstract

Consumption of milk and dairy products is considered one of the main routes of human exposure to *Mycobacterium avium* subsp. *paratuberculosis* (MAP). Quantitative data on MAP load in raw cows’ milk are essential starting point for exposure assessment. Our study provides this information on a regional scale, estimating the load of MAP in bulk tank milk (BTM) produced in Emilia‐Romagna region (Italy). The survey was carried out on 2934 BTM samples (88.6% of the farms herein present) using two different target sequences for qPCR (f57 and IS900). Data about the performances of both qPCRs are also reported, highlighting the superior sensitivity of IS900‐qPCR. Seven hundred and eighty‐nine samples tested MAP‐positive (apparent prevalence 26.9%) by IS900 qPCR. However, only 90 of these samples were quantifiable by qPCR. The quantifiable samples contained a median load of 32.4 MAP cells mL ^−1^ (and maximum load of 1424 MAP cells mL ^−1^). This study has shown that a small proportion (3.1%) of BTM samples from Emilia‐Romagna region contained MAP in excess of the limit of detection (1.5 × 10^1^ MAP cells mL ^−1^), indicating low potential exposure for consumers if the milk subsequently undergoes pasteurization or if it is destined to typical hard cheese production.

## Introduction

*Mycobacterium avium* subsp. *paratuberculosis* (MAP) is the etiological agent of paratuberculosis (Johne's disease), a chronic gastrointestinal inflammation affecting both domestic and wild ruminants. Due to some clinical similarities, paratuberculosis has been linked with Crohn's disease in humans (Hermon‐Taylor and Bull 2002); however, the role, if any, of MAP in Crohn's disease has not been definitively demonstrated (Chiodini et al. 2012). More recently, MAP has also been linked with the development of other human diseases, like multiple sclerosis, type I diabetes, and others (Scanu et al. 2007; Sisto et al. 2010; Cossu et al. 2011; Dow 2012; Atreya et al. 2014).

Cows’ milk consumption is considered to be one of the major potential routes of human exposure to MAP (Gill et al. 2011). Previous studies have shown that pasteurization and other technological processes can lead to a significant decrease of MAP in dairy products (Eltholth et al. 2009; Gill et al. 2011; Okura et al. 2012). The probability of MAP survival after milk pasteurization is strictly related to the MAP load in raw milk. For this reason, the availability of this information is critical to estimate the exposure potential of consumers of raw milk, and to address the survival ability of MAP in pasteurized milk and dairy products. This is particularly important in the evaluation of the possibility that MAP survives milk pasteurization. Assuming that this process can decrease viable MAP concentration by 4–7 log_10_ (Gill et al. 2011), a high load of MAP in raw milk could lead to contaminated dairy products. Accordingly, many reports have described the presence of viable MAP in raw and pasteurized cows’ milk, cheese, and other dairy products at retail level, posing a potential risk for the consumers (Weber et al. 2008; Eltholth et al. 2009; Okura et al. 2012).

MAP concentration in milk of infected cows due to direct excretion has been estimated to be less than 100 CFU mL^−1^ for clinical cows and 2–8 CFU 50 mL^−1^ for asymptomatic cows (Sweeney et al. 1992; Giese and Ahrens 2000). However, MAP‐infected cows can excrete the bacterium in their feces in very high numbers (Cocito et al. 1994), and it has been estimated that a mean of 59 mg of feces/dirt per liter of milk can be transmitted during milking of soiled udders (Vissers et al. 2007). Thus, naturally occurring concentrations of MAP in raw milk could be substantially increased by fecal contamination during milking (Rademaker et al. 2007; Atreya et al. 2014).

Essentially, two kinds of test are used to ascertain the presence of MAP in bulk tank milk (BTM) – polymerase chain reaction (PCR) methods and cultural methods. The former is credited with higher detection sensitivity in milk (Eltholth et al. 2009; Okura et al. 2012). Several studies have investigated the presence of MAP DNA in BTM (see Eltholth et al. 2009; Gill et al. 2011; Okura et al. 2012), but only a few have estimated the concentration of MAP present by quantitative PCR (Herthnek et al. 2008; Slana et al. 2008, 2009; Grant and Foddai 2014). More recently, using a novel assay involving peptide‐mediated magnetic separation and a phage amplification (PMS–phage assay) to test BTM from Johne's affected herds, Grant and Foddai (2014) reported levels of viable MAP ranging from 18 to 695 MAP 50 mL^−1^ BTM. Data on MAP load in milk are the essential starting point for microbiological exposure assessment (Gardner 2004) and the current scarcity of such data is underlined by a recent meta‐analysis (Okura et al. 2012). For this reason, the aims of this study were (1) to evaluate the performances of quantitative PCRs (qPCR) targeting IS900 and f57, and (2) to estimate the MAP load in BTM from almost all dairy herds in a northern Italian region (Emilia‐Romagna) through quantitative PCR data.

## Materials and Methods

### BTM DNA extraction

Fifty milliliters of BTM was centrifuged at 2500*g* for 15 min. The supernatant was discarded and the pellet was resuspended in 1 mL of PBS–Tween 20 (0.05%) and processed by PMS (Foddai et al. 2010; Serraino et al. 2014) using an automated magnetic capture system (BioSprint 96, Qiagen, Milan, Italy) in order to capture MAP cells. At the end of the PMS procedure, the magnetic beads were suspended in 300 *μ*L of sterile distilled water and then submitted to DNA extraction.

DNA was extracted from PMS‐captured MAP cells, following a previously described protocol (Jayarao et al. 2004), with some modifications. Briefly, 300 mg of acid‐washed glass beads of 150–212 *μ*m diameter (Sigma Aldrich, Milan, Italy) were added to the bead suspension obtained after PMS and subjected to bead beating in Tissue Lyser II (Qiagen, Milan, Italy) at 30 Hz for 10 min. After this mechanical lysis, 100 *μ*L of the sample were added to a 1.5‐mL tube containing 20 *μ*L of Proteinase K and 80 *μ*L of Chelex resin (Biorad, Milan, Italy), and incubated at 70°C for 10 min followed by 15 min at 95°C, then centrifuged at 12,000*g* for 5 min. Four microliters of the supernatant was used as DNA template for qPCR.

### Quantitative PCRs

Two already described qPCRs targeting IS900 (Donaghy et al. 2011) and f57 (Ricchi et al. 2014) sequences were employed in this study. f57 is present in only one copy in MAP genome, while IS900 is present in around 15–18 copies, which means the latter qPCR has potentially superior detection sensitivity. An internal positive control (TaqMan^®^ Exogenous Internal Positive Control, Life Technologies, Monza, Italy) was added to each PCR reaction, in order to detect possible false‐negative results due to PCR inhibition. A negative extraction sample was processed in parallel during each DNA extraction. These last samples, together with a negative and a positive PCR controls, were added to all qPCR runs in order to check each amplification reaction. All reactions were run on StepOne Plus qPCR system (Life Technologies) using TaqMan Universal Master Mix without UNG (Life Technologies). Raw data were processed by StepOne Software (version 2.3) (Life Technologies). According to minimum information for publication of quantitative real‐time PCR experiments (MIQE) guidelines (Bustin et al. 2009), information about the analytical sensitivity and efficiency of both qPCRs (IS900 and f57) and linear range are available in Figure S1, while information about the analytical specificity and reagents concentrations are reported in the original papers (Donaghy et al. 2011; Ricchi et al. 2014).

### Plasmid standard generation

The plasmid standards used in this study harbored inserts obtained using already described f57 primers (Ricchi et al. 2014) and IS900 primers (Millar et al. 1995). The amplicons were inserted into the pCR2.1‐TOPO vector (Life Technologies), the DNA was extracted using QIAprep Spin Miniprep Kit (Qiagen), quantified by 260 nm and the number of plasmid copies was calculated according to Lee et al. (2006).

### Determination of qPCR limit of detection of the PMS‐qPCRs

The limit of detection (LOD) in this study was defined as the lowest copy number that gives a detectable PCR amplification product at least 95% of the times, that is, LOD_95%_ (Burns and Valdivia 2008). A MAP reference strain (ATCC 19698) and a MAP field isolate (IZSLER 76/13) were used to determine the LOD of the qPCR methods. The field strain was originally isolated from cattle feces, and classified as Type C using PCR‐REA on IS1311 (Marsh et al. 1999), at the Italian National Reference Centre for Paratuberculosis. MAP suspensions were prepared according to Logar et al. (2012). Briefly, colonies from solid cultures were harvested and resuspended in PBS with glass beads (diameter ca. 5 mm), then vortexed for 45 sec to break down the clumps. The optical density at 600 nm was adjusted to 0.7 and the absence of clumps confirmed by microscopic examination using a Burker chamber. The suspensions were 10‐fold serially diluted in PBS containing glass beads, with vortexing for 45 sec between dilution steps, then 100 *μ*L of each dilution were streaked onto plates of Herrold's Egg Yolk Agar with Mycobactin J 2 mg L^−1^ (HEYM) for the determination of MAP concentration expressed as CFU mL^−1^.

In addition, 300 *μ*L of each dilution were centrifuged at 8000*g* for 5 min, the supernatant was removed and the pellet was suspended in 300 *μ*L of sterile distilled water containing Fish Sperm DNA (50 ng *μ*L^−1^; Ambion, Monza, Italy). MAP cells were mechanically disrupted in Tissue Lyser II (Qiagen, Milan, Italy) for 10 min at 30 Hz in the presence of 300 mg of 150–212 *μ*m diameter acid‐washed glass beads (Sigma Aldrich, Milan, Italy) and then heated at 100°C for 20 min. After centrifugation at 16,000*g* for 5 min, 4 *μ*L of the supernatant was used as template for f57‐qPCR and absolute MAP quantity was determined through a calibration curve generated with plasmid standards, using primers and probe previously reported (Slana et al. 2008). MAP concentration, measured by f57‐qPCR, was expressed as MAP cells mL^−1^.

In order to produce milk samples with different MAP concentrations per mL of milk, 50 mL aliquots of MAP‐free BTM were spiked with the previously described MAP suspensions. Since the sensitivity of paratuberculosis tests is low, in order to have MAP‐free BTM, BTM was obtained from a single herd with a known negative history of paratuberculosis (internal breeding, ELISA, and culture assays repeatedly negatives over the past 5 year for all adult animals). The samples were mixed by vortexing and then centrifuged at 2500*g* for 15 min, the supernatant was discarded and the DNA extracted from the remaining pellets, as described earlier for BTM DNA extraction. To determine the LOD_95%_, 10 replicates of 50 mL of BTM containing concentrations of MAP cells ranging from approximately 10^1^ to 10^4^ MAP cells mL^−1^ were tested by qPCR. The values of LOD_95%_ for both qPCR methods applied were estimated through generalized linear models with binomial error distribution and logit link functions as recommended in the OIE Manual of Diagnostic Tests and Vaccines for Terrestrial Animals (Burns and Valdivia 2008; Anonymous [Link], [Link]). The statistical analyses were performed with R 3.2.0 (R Foundation for Statistical computing 2010) and library MASS.

Therefore, for samples having a MAP concentration above the LOD, that is, a concentration high enough to make those samples detectable with the specified confidence (i.e., 95% of the time in this study), the limit of quantification could be deemed reliable provided that the quantification cycle (Cq) of the second IS900‐qPCR (see below) falls within the interval covered by the plasmid standard calibration curves.

To correct the quantitative output of the qPCR for the efficiency of DNA extraction, we scaled the values obtained from qPCR using linear regression models. Specifically, we fitted two log‐linear models with input MAP cells concentration (amount of MAP cells used for the spiking of samples) as explanatory variable and experimental output MAP cells concentrations obtained with IS900 and f57 qPCR as response variables (amount of MAP cells recovered after qPCR) for both MAP strains used for the determination of the LOD_95%_.

### BTM testing

A qualitative and quantitative survey of MAP contamination of BTM from all bovine dairy herds in the Emilia‐Romagna Region of Italy was carried out from March to June 2013. In total, 2934 BTM samples were tested; 2880 samples derived from single herds, while the remaining 54 samples were derived from pools of 2–10 small dairy herds (for a total number of 3067 herds tested). The median number of cows in the pooled herds was 31, while the median number of cows in the pools derived from these herds was 123. The BTM samples were collected during the periodic (6 months turn around) surveillance survey for Brucellosis in the dairy herds by Regional Veterinary Health Services. The sampling represents 88.6% of the bovine dairy herds in the Emilia‐Romagna region. All BTM samples were stored at 4°C after collection and delivered to the testing laboratory within 24 h or eventually frozen at −20°C until the analysis, which in any case was carried out within 1 week. After DNA extraction, all samples were screened for the presence of MAP DNA with an IS900‐qPCR run in duplicate. In order to maximize the sensitivity of the IS900‐qPCR assay employed, milk samples were considered as positive if at least one of the duplicates showed a Cq value under the cutoff (<38 Cqs), which was chosen according to Donaghy et al. (2011). For samples in which both replicates showed a Cq value <38, the concentration of MAP cells present in the milk was quantified using the same IS900‐qPCR, by comparison with plasmid standards of known copy number according to Slana et al. (2008). For samples showing Cq values <36 on the initial IS900‐qPCR, a second quantification assay was performed using the f57‐qPCR, again including plasmid standard harboring f57 target sequence. A different Cq threshold was adopted taking into account the lower sensitivity of the f57‐qPCR than that targeting IS900.

## Results

### Validation of PMS‐qPCRs

**Figure 1:**
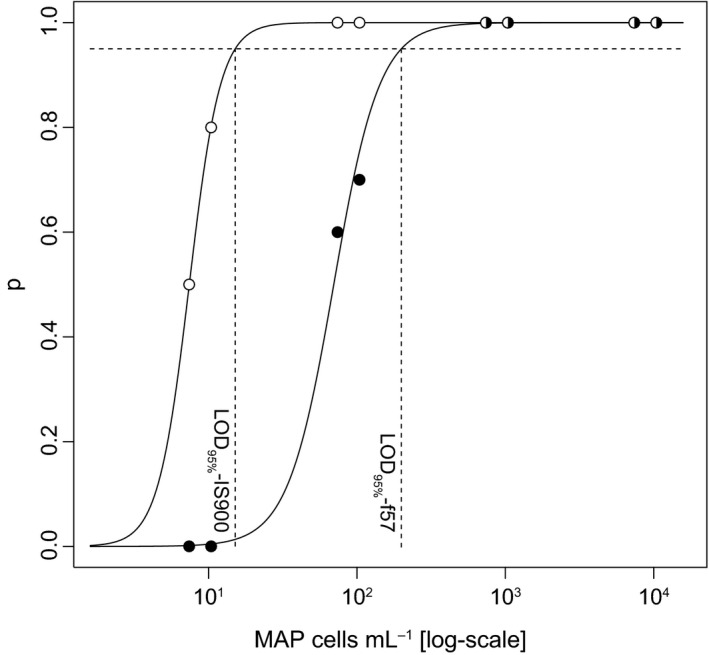
Probabilities of MAP detection through qPCRs derived from the serial dilutions of spiked milk (white circles: IS900, black circles: f57). The *x*‐axis represents the log_10_ of MAP cells mL^−1^ of milk and the *y*‐axis represents the probability of detection. The curves represent the best generalized linear models to the data points. The horizontal dashed lines represent the 95% probability of detection of the two qPCRs and the vertical dashed lines represent the corresponding limit of detection of 95% values for IS900 and f57 targets. MAP, *Mycobacterium avium* subsp. *paratuberculosis*.

Based on data reported in Table 1, and according to the generalized linear model, the LOD_95%_ of the two PMS‐qPCR methods were assessed to be 1.5 × 10^1^ MAP cells mL^−1^ for IS900‐qPCR and 2.0 × 10^2^ MAP cells mL^−1^ for f57 qPCR (see Fig. 1), corresponding to 7.5 × 10^2^ MAP cells per 50 mL of milk and 10^4^ MAP cells per 50 mL of milk, respectively. Notably, the concentration of the input of MAP, which was used during the spiking of negative samples for the determination of the LODs, when measured by cultural plate counting, was approximately 10‐ to 100‐fold lower than that obtained by qPCRs (Table 1). The efficiency of MAP recovery from MAP spiked samples was similar for both qPCR targets, with median values of 17% for IS900 and 19% for f57 (see Table 1). Moreover, the efficiency was quite consistent across the different concentrations. In fact, the log‐linear regressions implemented to correct the quantitative output of the qPCR for the efficiency of DNA extraction showed values of slope very close to the unity (1.06 ± 0.036 for IS900, 1.005 ± 0.042 for f57).

**Table 1:** Experimental output of MAP cells mL^−1^ of milk obtained by IS900‐qPCR and f57‐qPCR after spiking negative milk samples with known amount of MAP

MAP strain	Input MAP cell concentration (f57‐qPCR)^a^	Input MAP cell concentration (culture)^b^	Experimental output of MAP cells by IS900 qPCR^c^			Experimental output of MAP cells by f57 qPCR d		
			Mean^e^	SD	Signal ratio^f^	Mean^e^	SD	Signal ratio^f^
ATCC19698	1.0 × 10^4^	6.5 × 10^3^	1.3 × 10^3^	2.7 × 10^2^	10/10	1.8 × 10^3^	3.1 × 10^2^	10/10
	1.0 × 10^3^	6.5 × 10^2^	2.5 × 10^2^	12.0 × 10^1^	10/10	2.5 × 10^2^	12.8 × 10^1^	10/10
	1.0 × 10^2^	6.5 × 10^1^	2.7 × 10^1^	2.1 × 10^1^	10/10	3.1 × 10^1^	2.0 × 10^1^	7/10
	1.0 × 10^1^	—	3.4 × 10^0^	3.7 × 10^0^	8/10	—	—	0/10
IZSLER76/13	0.7 × 10^4^	2.3 × 10^2^	2.4 × 10^3^	5.3 × 10^2^	10/10	1.9 × 10^3^	2.2 × 10^2^	10/10
	0.7 × 10^3^	2.3 × 10^1^	1.8 × 10^2^	10.3 × 10^1^	10/10	1.2 × 10^2^	6.6 × 10^1^	10/10
	0.7 × 10^2^	—	1.5 × 10^1^	7.9 × 10^0^	10/10	1.8 × 10^1^	1.5 × 10^1^	6/10
	0.7 × 10^1^	—	0.5 × 10^0^	0.4 × 10^0^	5/10	—	—	0/10

a Number of MAP cells mL^−1^ in the pure cultures/suspensions used for the spiking of milk samples evaluated by f57 qPCR.

b Number of MAP cells mL^−1^ in the pure cultures/suspensions used for the spiking of milk samples evaluated on culture in Herrold's Egg Yolk Agar with Mycobactin‐ANV plates in duplicate by seeding 100 *μ*L and expressed as CFU mL^−1^.

c Number of MAP cells mL^−1^ recovered after DNA isolation by IS900 qPCR, the value was divided by 15 to obtain real number of MAP cells.

d Number of MAP cells mL^−1^ recovered after DNA isolation by f57 qPCR.

e The mean values correspond to the absolute number of MAP cells mL^−1^.

f Number of positive replicates/total number of replicates.

### BTM survey results

**Figure 2:**
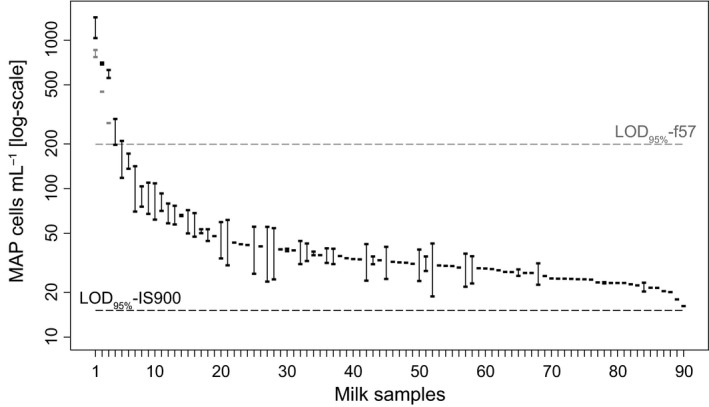
Estimated MAP concentration in the quantifiable bulk tank milk samples obtained through qPCR (targets: IS900 in black, f57 in gray). Dashed lines represent the limit of detection of 95% for IS900 (black) and f57 (gray) targets. Rectangles represent the point estimates of MAP cells mL^−1^ for a given sample. Vertical lines represent samples with two quantifiable reactions. MAP, *Mycobacterium avium* subsp. *paratuberculosis*.

Of the 2934 BTM samples tested, 789 (26.9% of the total) were positive by IS900‐qPCR test, but only 279 were suitable for quantification according to positive call for both replicates (see Materials and Methods section). After the second IS900‐qPCR aimed at the quantification of the MAP concentration in milk, 90 samples showed a MAP concentration above the LOD_95%_. The distribution of MAP concentrations in the 90 samples above the LOD_95%_ is shown in Figure 2 (in black). For only 41 samples, both technical replicates gave a quantifiable result (i.e., both replicates within the limits of the calibration curves), while for the rest of the samples, only one technical replicate provided a quantifiable result. In general, for samples with MAP concentration lower than ~50 cells mL^−1^, a reliable quantification for both technical replicates was not always possible. The distribution of MAP concentrations in the survey samples was very skewed, with the minimum and maximum observed concentrations of 16 cells mL^−1^ and 1424 cells mL^−1^, respectively, and the bulk of samples with values below 50 MAP cells mL^−1^. Overall, the median value of MAP in the quantifiable samples was 32.4 cells mL^−1^ with IS900‐qPCR.

Finally, only three of the above‐mentioned samples were quantifiable by f57‐qPCR (Fig. 2 in gray), with values very similar to those obtained by IS900‐qPCR. In this case, only one sample was quantifiable from both the technical replicates, confirming the lower sensitivity of f57‐qPCR compared to IS900‐qPCR.

## Discussion

The role of MAP as a potential zoonotic agent remains controversial and no definitive information regarding the health risk posed by consumption of raw milk contaminated by MAP is available, essentially because it is still uncertain if MAP per se can be related to the pathogenesis of Crohn's disease or any other human pathological conditions (Momotani et al. 2012). Nevertheless, as mentioned by Collins (2011), assessing consumers’ exposure to this potential zoonotic hazard appears to be prudent. This is particularly important considering that milk is generally consumed by children, who could be exposed to MAP via contaminated milk in the early stages of their life.

As expected, due to the different number of targeted copies, the PMS‐qPCR assays presented in this study showed a LOD_95%_ of 1.5 × 10^1^ MAP cells mL^−1^ for IS900‐qPCR and 2.0 × 10^2^ MAP cells mL^−1^ for f57 qPCR, as evaluated by logistic regressions. For this reason, IS900‐qPCR was the method used to test the survey samples in this study. Our data show that cultural methods for the evaluation of MAP cells used for the spiking of negative samples underestimate the true number of MAP cells present in the sample by around one or two log_10_ in comparison to qPCR (see Table 1), essentially confirming some previous observations (Herthnek et al. 2008; Elguezabal et al. 2011; Kralik et al. 2012). This seems to be due to the tendency of MAP to form clumps and to the death and/or possible dormancy of some MAP cells (Elguezabal et al. 2011) that remain detectable by qPCR but not by culture. Due to this difference, and since we used a PCR assay, we refer to the MAP load of the milk samples as number of cells mL^−1^ rather than CFU mL^−1^.

Data on the apparent prevalence of MAP in BTM‐positive samples detected by IS900‐PCR are extremely variable due to the different target populations and analytical methods employed. In Europe, a study from U.K. showed an apparent prevalence of 8% (Grant et al. 2002), while other surveys carried out in Ireland (O'Reilly et al. 2004), Denmark (Herthnek et al. 2008), Switzerland (Corti and Stephan 2002), Austria (Metzger‐Boddien et al. 2006), Cyprus (Slana et al. 2009), and Czech Republic (Slaná et al. 2009) showed higher apparent prevalence values, ranging from 13% (Ireland and Denmark) to 34% (Czech Republic). Also, the variability is very high when countries outside Europe are considered, for example, Brazil (0%, Carvalho et al. 2009), Iran (11%, Haghkhah et al. 2008), USA (27%, Jayarao et al. 2004), and Mexico (100%, Favila‐Humara et al. 2010).

In a recent meta‐analysis (Okura et al. 2012), the apparent prevalence of MAP in BTM samples on the basis of IS900‐PCR was assessed to be around 30% (16–49%, 95% CI), a value very similar to that obtained in this study (26.9%). However, the same meta‐analysis underlined that despite the large number of studies regarding the detection of MAP in milk (also reviewed by Eltholth et al. 2009 and Gill et al. 2011), very few studies have addressed the quantification of MAP in BTM samples (Okura et al. 2012). The few studies in which quantification has been attempted reported that the concentration of MAP in BTM rarely exceeded 100 MAP cells per mL of milk. In particular, in Denmark it has been shown to range from “a few cells” to 200 per mL of milk (Herthnek et al. 2008), while in Cyprus it ranged from “a few cells” to “tens of organisms” per mL of milk (Slana et al. 2009). Consistent with these results and according to our criteria, only 90 samples (3.1% of total samples analyzed) resulted quantifiable and only seven (0.2%) exceeded 100 MAP cells mL^−1^. The load of MAP in positive quantifiable BTM samples ranged from 16 to 1424 MAP cells mL^−1^ (median 32.4 cells mL^−1^). Notably, in our first screening, we detected 789 positive samples; however, according to our scheme of interpretation, we attempted quantification with the same IS900‐qPCR for only 279 samples that had both replicates positive and Cq <38. Interestingly, after the second analysis, only 90 samples gave a quantifiable result (at least one replicate falling within the calibration curve). This latter finding underlines the low amount of MAP DNA present in the majority of the positive BTM samples, near to the limit of detection of the assay.

To the best of our knowledge, only the study carried out in Switzerland (Corti and Stephan 2002) analyzed a number of samples from different areas of Switzerland comparable to our sample size (1384 against 2934 samples). However, the aim of that study was not to determine the load of MAP present in BTM, but simply presence of MAP. In contrast, our study analyzed 2934 BTM samples from almost all (~90%) dairy herds in the Emilia‐Romagna region, supplying information about both apparent prevalence and load of MAP in milk produced in this region. Overall, our results suggest that in 97% (2844 of 2934) of BTMs herein tested from the Emilia‐Romagna region of Italy, MAP is either absent or present below a concentration of 15 cells mL^−1^ (the LOD_95%_ of PMS‐IS900‐qPCR). The load of MAP in positive BTM estimated by our study suggests that human exposure related to milk consumption can be deemed to be limited considering that subsequent pasteurization of this milk should decrease the number of viable MAP by 4–7 log_10_ (Weber et al. 2008). However, 90% of the milk produced in the Emilia‐Romagna region is not submitted to pasteurization, but is destined for the production of hard cheese (e.g., Parmigiano Reggiano and Grana Padano), being heated at 53–56°C for 30–70 min. Nonetheless, the risk of human exposure to MAP via consumption of these cheeses would still be considered limited because preliminary results of MAP survival challenge tests in these products showed a reduction in the concentration of MAP during the creaming phase by 1 log_10_ (Cammi et al. 2014). A further reduction during the subsequent ripening phase (at least 9–12 months) has also been observed (Cammi, unpublished data), indicating extremely low likelihood of MAP survival by the time these products go on sale.

Nevertheless, considering (1) the high regional apparent prevalence of MAP‐positive BTMs; (2) the finding of some highly contaminated samples; (3) the presence of 172 vending machines which sell raw milk ( http://www.milkmaps.com) in the regional territory; and (4) the results of previous studies reporting the survival of MAP after pasteurization processes (Gill et al. 2011), our analysis suggests that although the risk for humans linked to the consumption of milk and dairy products herein produced seems to be limited, some concern for consumers cannot be excluded.

In order to decrease the presence of MAP in BTM, the application of control programs aimed at reducing the number of MAP‐infected cows in dairy herds is advisable. Particular attention should be given to identifying and removing cows showing clinical symptoms of Johne's disease, which are likely to shed the highest levels of MAP directly into their milk and which mainly contribute to the spread of MAP to the environment. Moreover, because environmental MAP contamination may also play a significant role in the final load of MAP in BTM, control programs should also focus on improvement of hygienic conditions at farm level, with the aim of reducing the extent of udder contamination by infected feces.

## Conflict of Interest

None declared.

## Supporting information


**Figure S1.** (A) The graph shows the analytical sensitivity (hypothetical optimal condition) of IS900 and f57‐qPCRs expressed as pg of pure MAP DNA, ranging from 10^−3^ to 10^1^ pg of MAP DNA per tube reaction. Each point represents the mean values ± SD of three different experiments in quadruplicated. Efficiency was evaluated with the equation: Efficiency = 10^−1/slope−1^. Purified MAP DNA was kindly provided by Dr. Karren Plain. *10 of the 12 positive calls, ^+^1 of the 12 positive calls. (B) The graphs show the linear range of IS900 and f57‐qPCRs relative to milk samples spiked with ATCC 19698 and IZLSER 76/13 strains, respectively. DNA was extracted according to the procedure described in the article. Each point represents the mean values ± SD of five replicates. Efficiency was evaluated with the equation: Efficiency = 10^−1/slope−1^. *4 of the 5 positive calls, ^#^1 of the 5 positive calls.
