# Supplementary material for: Estimation of Mycobacterium avium subsp. paratuberculosis load in raw bulk tank milk in Emilia‐Romagna Region (Italy) by qPCR
Source: Microbiologyopen. 2016 Mar 17;5(4):551–9. doi: 10.1002/mbo3.350 (PMC4985589; doi:10.1002/mbo3.350)
Supplement: Supplementary file 1 — Figure S1. (A) The graph shows the analytical sensitivity (hypothetical optimal condition) of IS900 and f57‐qPCRs expressed as pg of pure MAP DNA, ranging from 10−3 to 101 pg of MAP DNA per tube reaction. Each point represents the mean values ± SD of three different experiments in quadruplicated. Efficiency was evaluated with the equation: Efficiency = 10−1/slope−1. Purified MAP DNA was kindly provided by Dr. Karren Plain. *10 of the 12 positive calls, +1 of the 12 positive calls. (B) The graphs show the linear range of IS900 and f57‐qPCRs relative to milk samples spiked with ATCC 19698 and IZLSER 76/13 strains, respectively. DNA was extracted according to the procedure described in the article. Each point represents the mean values ± SD of five replicates. Efficiency was evaluated with the equation: Efficiency = 10−1/slope−1. *4 of the 5 positive calls, #1 of the 5 positive calls. [file MBO3-5-551-s001.pdf]

Figure 1 Supplemental Material

a)

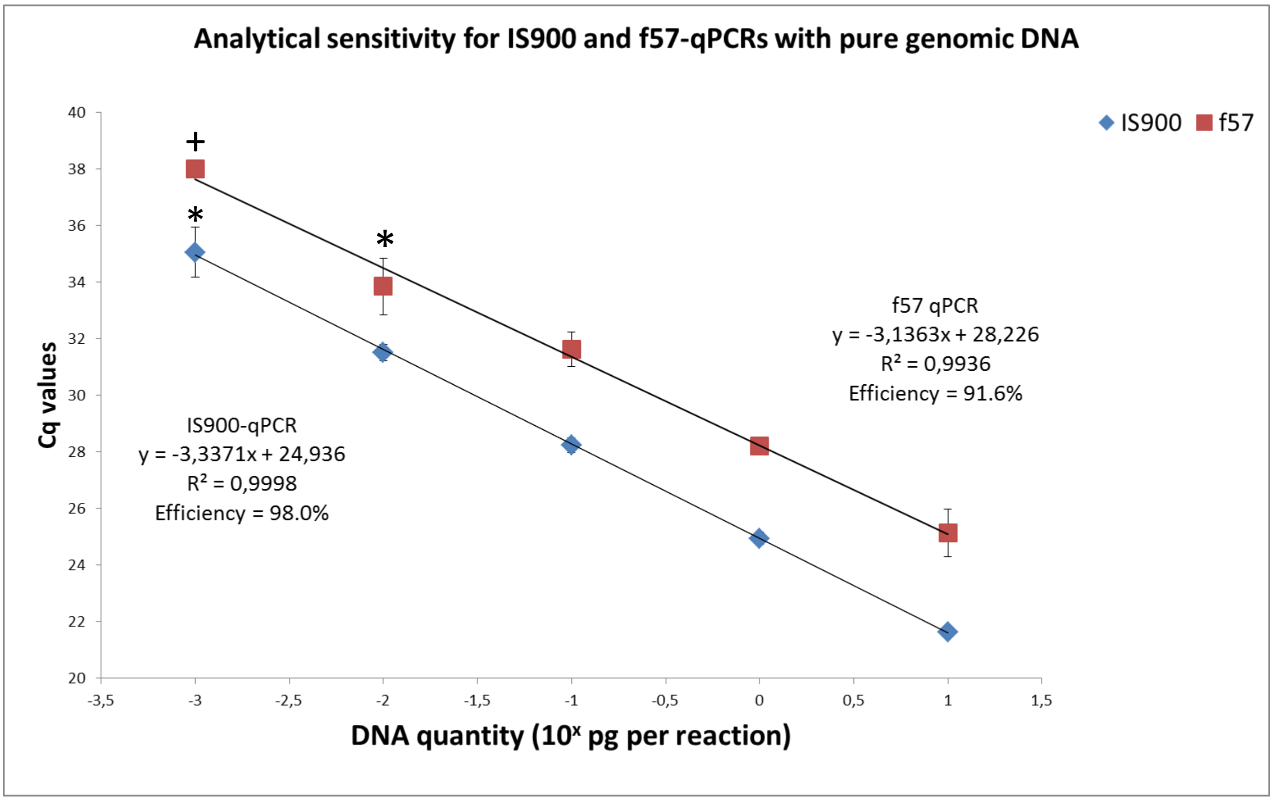

The graph shows the analytical sensitivity (hypothetical optimal condition) of IS900 and f57-qPCRs expressed as *pg* of pure MAP DNA, ranging from 10^{-3} to 10^{1} *pg* of MAP DNA *per* tube reaction. Each point represents the mean values ±SD of three different experiments in quadruplicated. Efficiency was evaluated with the equation Efficiency = $10^{-1/slope}$-1. Purified MAP DNA was kindly provided by Dr. Karren Plain.

* 10 out of 12 positive calls

$^{+}$ 1 out of 12 positive calls

b)

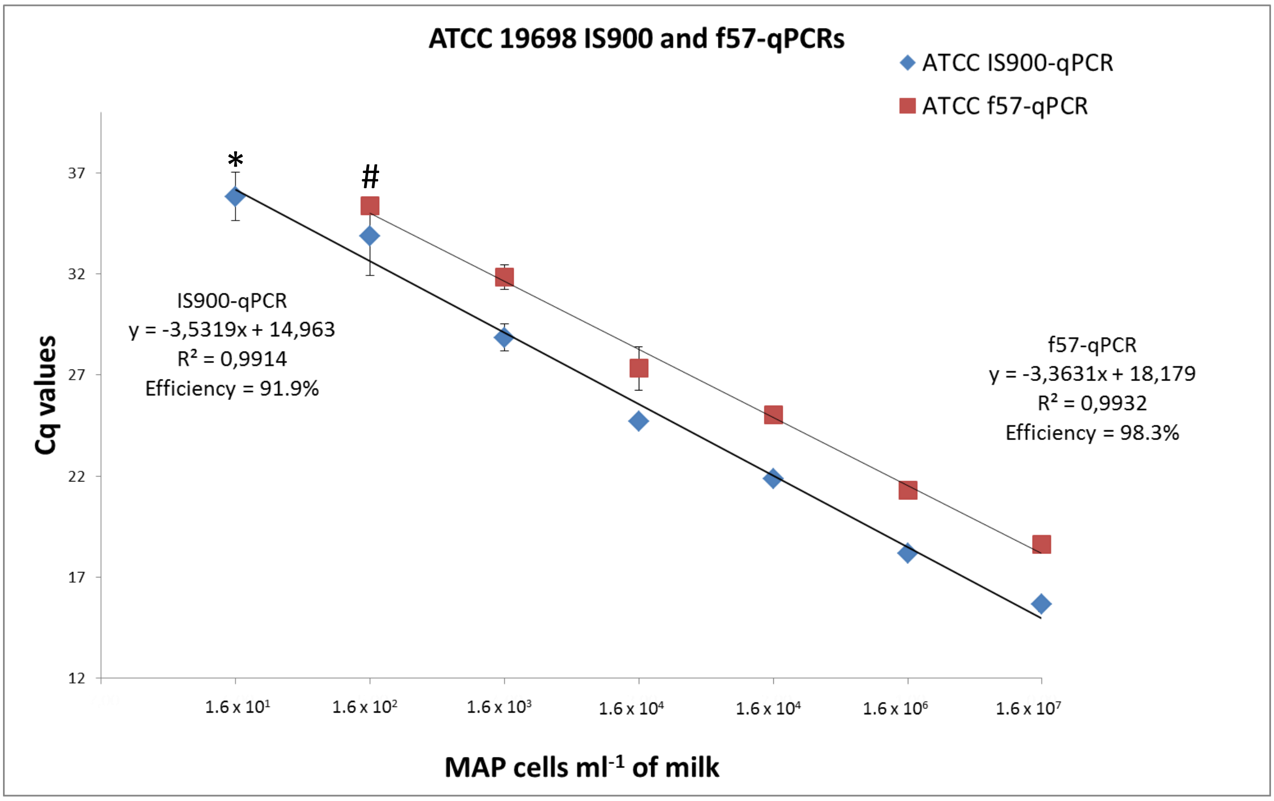

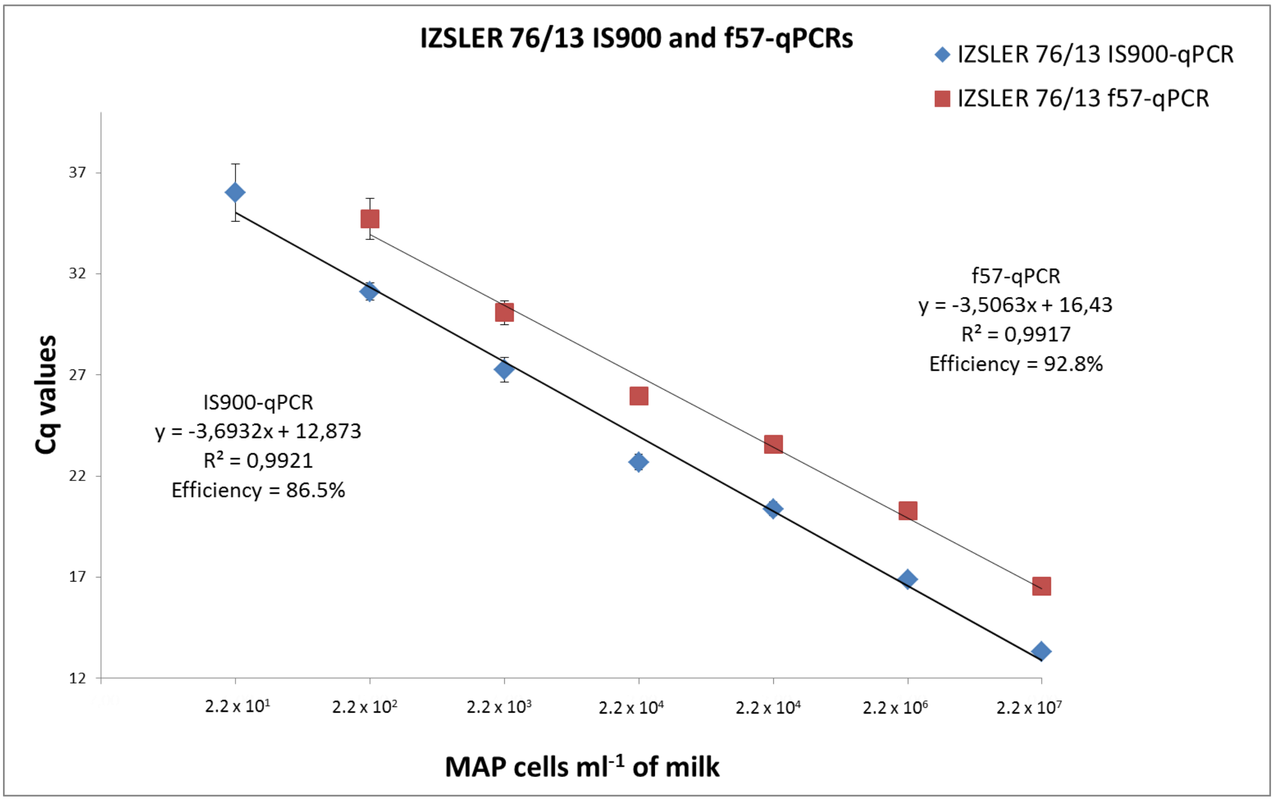

The graphs show the linear range of IS900 and f57-qPCRs relative to milk samples spiked with ATCC 19698 and IZLSER 76/13 strains respectively. DNA was extracted according to the procedure described in the paper. Each point represents the

mean values ±SD of five replicates. Efficiency was evaluated with the equation Efficiency = $10^{-1/slope}$-1.

* 4 out of 5 positive calls

[#] 1 out of 5 positive calls
